# Efficiency Optimization of CRISPR/Cas9-Mediated Targeted Mutagenesis in Grape

**DOI:** 10.3389/fpls.2019.00612

**Published:** 2019-05-16

**Authors:** Fengrui Ren, Chong Ren, Zhan Zhang, Wei Duan, David Lecourieux, Shaohua Li, Zhenchang Liang

**Affiliations:** ^1^Beijing Key Laboratory of Grape Sciences and Enology, Laboratory of Plant Resources, Institute of Botany, Chinese Academy of Sciences, Beijing, China; ^2^College of Life Sciences, University of Chinese Academy of Sciences, Beijing, China; ^3^EGFV, Bordeaux Sciences Agro, INRA, Université de Bordeaux, Villenave d’Ornon, France; ^4^Sino-Africa Joint Research Center, Chinese Academy of Sciences, Wuhan, China

**Keywords:** CRISPR/Cas9, optimization, GC content, grape, gene expression, gene editing efficiency

## Abstract

Clustered regularly interspersed short palindromic repeats (CRISPR)/Cas system is an efficient targeted genome editing method. Although CRISPR/Cas9-mediated mutagenesis has been applied successfully in grape, few studies have examined the technique’s efficiency. To optimize CRISPR/Cas9 editing efficiency in *Vitis vinifera*, we surveyed three key parameters: GC content of single guide RNA (sgRNA), variety of transformant cells used, and *SpCas9* expression levels in transgenic cell mass. Four sgRNAs with differing GC content were designed to target exon sites of the *V. vinifera* phytoene desaturase gene. Suspension cells of ‘Chardonnay’ and ‘41B’ varieties were used as the transgenic cell mass. Both T7EI and PCR/RE assays showed that CRISPR/Cas9 editing efficiency increases proportionally with sgRNA GC content with 65% GC content yielding highest editing efficiency in both varieties. Additionally, gene editing was more efficient in ‘41B’ than in ‘Chardonnay.’ CRISPR/Cas9 systems with different editing efficiency showed different *SpCas9* expression level, but compared with GC content of sgRNA, *SpCas9* expression level has less influence on editing efficiency. Taken together, these results help optimize of CRISPR/Cas9 performance in grape.

## Introduction

Targeted genome editing (TGE) using site-specific nucleases (SSNs) is a popular technique for studying gene function and new traits (Lee et al., 2016). These powerful SSN tools introduce targeted DNA double-strand breaks to trigger DNA repair pathways involving either non-homologous end-joining (NHEJ) or homologous recombination (HR) (Symington and Gautier, 2011). Gene editing is performed with zinc-finger nucleases (ZFNs), transcription activator-like effector nucleases (TALENs), or clustered regulatory interspaced short palindromic repeats (CRISPR)/CRISPR-associated protein 9 (Cas9) system (CRISPR/Cas9). However, designing appropriate constructs for the first two techniques is complex and costly, leading to a preference for the CRISPR/Cas (Lozano-Juste and Cutler, 2014). Of the three types of CRISPR/Cas systems (I, II, and III), type II (Cas9 protein) is the most popular because it is highly effective in facilitating RNA-guided SSNs (Cong et al., 2013; Wu et al., 2014). CRISPR/Cas9 has been used for TGE in a wide variety of animals and plants. Examples of the former include mammalian cells, zebrafish embryos (Hwang et al., 2013), and mice (Yang et al., 2013). Examples of the latter include *Arabidopsis* (Feng et al., 2013; Li et al., 2013), tobacco (Shan et al., 2014), wheat (Shan et al., 2014), grape (Ren et al., 2016), sorghum (Jiang et al., 2013), maize (Chen and Gao, 2014), soybean (Jacobs et al., 2015), and tomato (Brooks et al., 2014; Pan et al., 2016). Despite this popularity, the CRISPR/Cas system still has unresolved limitations, such as off-target mutagenesis, low editing efficiency, variation in specificity, the presence of protospacer adjacent motif (PAM) sequences and recalcitrant sgRNA/targets.

Grapes (*Vitis vinifera*) are one of the most important and economically valuable fruit crops worldwide. Due to ongoing climate change, there is also an increasingly urgent need for breeding improvement. Although whole-genome sequence is available for grapevine (*V. vinifera* ‘Pinot Noir’) (Jaillon et al., 2007), functional genomics has been hampered by the lack of stable and efficient genetic transformation protocols. Fortunately, a recent study found success in using CRISPR/Cas9 for targeted mutagenesis in grapevine (Ren et al., 2016). Subsequently, the CRISPR/Cas9 system was also successfully used to target the grapevine genes *VvWRKY52* (a transcription factor) (Wang et al., 2018) and *VvPDS* (phytoene desaturase) (Nakajima et al., 2017). Nevertheless, editing efficiency remains an area that requires further improvement.

The development of a robust transformation system suitable for a wide range of cultivars is necessary for improving CRISPR/Cas9 efficiency in grape. Currently, some grape cultivars are recalcitrant to *Agrobacterium*-mediated transformation, though this method is effective in several others (Iocco et al., 2001). Furthermore, agrotransformation is unreliable and inefficient in grape compared with model plants, such as *Arabidopsis thaliana* and *Nicotiana benthamiana*. Desired transgenic grape lines thus take much longer to develop than in traditional model plants. These between-species differences are due to numerous parameters, including *Agrobacterium* strain, culture medium, antibiotic concentration, and temperature. Clearly, there is a pressing need to understand the exact factors that influence efficiency of CRISPR/Cas9 genome editing in grapevine.

In this study, we investigated three parameters that can potentially be optimized to improve TGE in grape. The first factor is the GC content of sgRNA used to target our gene of interest (phytoene desaturase, *VvPDS*). Phytoene desaturase is part of the carotenoid biosynthetic pathway, and its reduction or loss results in a photobleaching phenotype due to chlorophyll photooxidation (Qin et al., 2007). Therefore, plants carrying functionally disrupted PDS have distinct albino and dwarf morphologies that are easily identifiable from wild-type. The second is cultivar used for transformation, while the last is *SpCas9* gene expression in transgenic cell masses (CMs). By analyzing the relationship between these key parameters and determining CRISPR/Cas9 efficiency under different conditions, we provided the basis for a high efficiency CRISPR/Cas9-mediated genome editing protocol. Our findings should help to promote the development of functional genomics and breeding improvement in grape.

## Materials and Methods

### Plant Materials

‘Chardonnay’ (*V. vinifera* L.) and ‘41B’ (*V. vinifera* ‘Chasselas’ × *V. berlandieri*) were derived from induced embryogenic calli. The former was cultured in 100 mL flasks filled with 25 mL of liquid CSM medium [MS basal medium supplemented with 0.5 g/L glutamic acid, 1 mg/L 2-naphthoxyacetic acid (NOA), 5.0 mL/L glycerol, 20 g/L maltose, pH 5.8]. The latter was cultivated in liquid GM medium [MS medium 1/2 Macro, with 1 g/L *N*-Z-Amine A, 1 mg/L 2-naphthoxyacetic acid (NOA), 4.6 g/L glycerol, Maltose 18 g/L, 1 mL/L Vitamins GMox1000, pH 5.8]. Suspension cells were shaken at 117 rpm and 27°C in the dark. Cells were sub-cultured every 7 days.

### Extraction of Genomic DNA

Genomic DNA was extracted from wild-type (WT) and resistant CM using CTAB (Zhang et al., 2016). First, 700 μL pre-heated CTAB buffer was added into 100 mg CM ground in liquid nitrogen. The mixture was then incubated at 65°C for 20 min before 700 μL chloroform was added to each sample. After centrifugation at 12,000 rpm for 5 min, the supernatant was transferred to a new tube, followed by the addition 500 μL isopropanol and incubation at 4°C for 30 min. The solution was centrifuged again at 12,000 rpm for 10 min to separate out genomic DNA. The DNA pellet was washed with 500 μL of 70% ethanol, and dissolved in 100 μL ddH_2_O to measure via spectrophotometer.

### Cloning of *VvPDS* Exon

The *VvPDS* exon was amplified from genomic DNA of both cultivars using High-Fidelity DNA polymerase KOD-plus Neo (TOYOBO, Japan). Specific primers (Supplementary Table S1) were designed based on the homologous gene VIT_09s0002g00100 from the EnsemblPlants^1^. Thermocycling conditions were as follows: 95°C for 5 min; 45 cycles of 95°C for 10 s, 57°C for 30 s, and 68°C for 30 s; followed by a final extension at 68°C for 5 min. The PCR product was cloned into the pClone007 Simple Vector (TSINGKE), and around five clones were sequenced.

### Design of sgRNA and Assembly of CRISPR/Cas9 Construct

CRISPR/Cas9 target sites were designed from verified sequences with the online tools Grape-CRISPR Database^2^. Four target sites (Table 1) were selected for designing target sgRNAs based on their GC content, location in the gene, and off-target possibilities. The pP1C.4 vector is the backbone of the CRISPR/Cas9 vector carrying plant-optimized *Streptococcus pyogenes* Cas9 protein-coding gene (Genloci, China). To obtain the AtU6-sgRNA cassette, reverse primers (Supplementary Table S1) comprising of 20 bp sgRNA sequences and an adaptor were used. To amplify the AtU6 promoter fragment, PCR was used to combine the AtU6 promoter and sgRNA. Amplified AtU6-sgRNA fragments containing adaptors could be inserted into the homologous sites of the linearized vector by the homologous recombination method.

**Table 1:** Summary of the six selected sgRNA.

Name	Position	Sequence	GC content
sgRNACrP1	Exon2	TCAATTCAGATATGTTTCTG	30%
sgRNACr1	Exon4	TTTGTCTACTGCAAAATATT	25%
sgRNACr3	Exon7	GCCAGCAATGCTCGGAGGAC	65%
sgRNACr4	Exon6	TCAAATCGGCTGAATTCCCC	50%

### Growth, Transformation of Grape Suspension Cells

Grape cells were transformed using an *Agrobacterium tumefaciens* cocultivation method (Martinelli and Mandolino, 1994). Final plasmids were introduced into *A. tumefaciens* using the freeze-thaw method described by manufacturer protocols. The prepared *A. tumefaciens* suspension was inoculated with suspension cell cultures collected though a brief centrifugation. After that, for rapid selection, we transferred them into selective liquid CSM medium and liquid GM medium with 5 mg/L hygromycin until resistant CMs were generated. The transformation events with the same Cas9/sgRNA construction were repeated three times at the same time to keep the activity of grape suspension cells and *A. tumefaciens* consistent. The hygromycin and liquid medium needed to be replaced every 7 days.

### Identification of PCR-Generated Exogenous T-DNA Insertion

Specific primers for the hygromycin-resistance gene (Supplementary Table S1) were used for PCR with High-Fidelity DNA polymerase KOD-plus Neo (TOYOBO, Japan). The thermocycler was set at 95°C for 5 min; 34 cycles of 95°C for 10 s, 60°C for 30 s, and 68°C for 30 s; followed by a final extension at 68°C for 5 min. Amplicons were separated on an EtBr-stained agarose gel (1.0%), then cloned into the pclone007 vector for Sanger sequencing.

### Detection and Sequencing of Mutations in Transgenic CMs

Prepared genomic DNA of transgenic CMs was used as a template to amplify genomic fragments containing target sites. Specific primers (Supplementary Table S1) were designed to PCR-amplify a 300–600 bp product containing the target site. The PCR reaction was performed with High-Fidelity DNA polymerase KOD-plus Neo in a total volume of 50 μL at 95°C for 5 min; 45 cycles of 98°C for 10 s, 57°C for 30 s and 68°C for 30 s; followed by a final extension at 68°C for 5 min. Amplicons were cloned into pclone007. Approximately 10 clones were sequenced to search for the chimerism in CM.

### Identification of CRISPR/Cas9 Efficiency

Two strategies were selected to determine CRISPR/Cas9 system efficiency (Shan et al., 2014). For both assays, PCR was first performed following procedures described above. Amplicons of sgRNACr1-CM and sgRNACr4-CM lines were then digested with the appropriate, assay-specific enzymes, following manufacturer protocol. Bands were visualized with gel electrophoresis. The WT-CM line was used as a positive control. Band intensity (used for estimating indel frequency) was quantified in ImageJ. Three biological replicates were performed for each transgenic and WT line.

#### PCR/Restriction Enzyme (RE) Assay

This assay requires a RE site that can be disrupted by CRISPR/Cas9-induced mutations. Digestion with the restriction enzyme would then yield un-cleaved bands in PCR. The RE used on sgRNACr1-CM and sgRNACr4-CM amplicons were SspI and EcoRI (NEB, United States), respectively. An EtBr-stained 2.5% agarose gel was used to separate bands.

Indel frequency was calculated with the following formula:

Indel(%)=100%×a÷(a+b+c)

where a is intensity of the undigested PCR product, while b and c are intensities of the two digested products. Mean indel efficiency was calculated from the three replicates.

#### T7EI Assay

When the target gene does not include an appropriate RE site, the T7EI assay is a better option for assessing CRISPR/Cas9 efficiency. The T7EI nuclease can digest CRISPR/Cas9-induced mismatched dsDNA, leaving behind WT and mutant homoduplexes. Amplicons of sgRNACr3-CM and sgRNACrP1-CM were digested with T7EI enzyme (NEB, United States). An EtBr-stained 3.0% agarose gel was used for separating bands.

The indel frequency was calculated from the following formula:

$$\text{Indel}(100\%)=100\times \left(1-\sqrt{1-\frac{b+c}{a+b+c}}\right)$$

where a is intensity of undigested PCR product, while b and c are intensities of the two digested products. Mean indel efficiency was calculated from the three independent replicates.

### Quantitative RT-PCR Analysis

Total RNA was extracted from ‘Chardonnay’ and ‘41B’ CMs using TRIzol reagent (Invitrogen, United States). Complementary DNA was synthesized from 1 μg of RNA using the HiScript Q RT SuperMix for qPCR (+ gDNA wiper) kit (Vazyme, China) following the manufacturer-provided protocol. Quantitative RT-PCR was performed in a final volume of 20 μL on a CFX96 Real-Time System (Bio-Rad, United States), with *SpCas9* specific primers (Supplementary Table S1). Grape Actin1 (AY680701) was used as an internal control. Relative expression level was calculated using the 2-ΔΔCT method. All experiments were performed with three biological replicates and three technical replicates.

## Results

### Selection of sgRNA for Constructing *VvPDS* and CRISPR/Cas9 Expression Vectors

**FIGURE 1:**
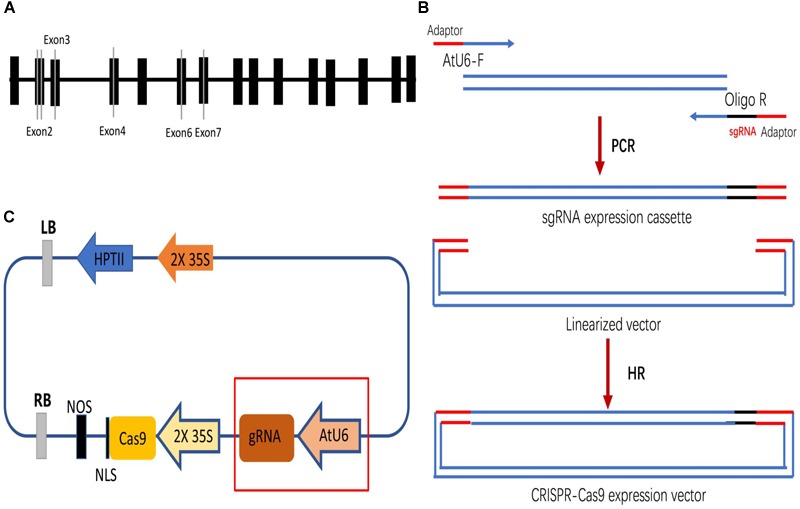
Selecting target sites in the *VvPDS* gene and constructing Cas9/sgRNA expression vectors. **(A)** Four sgRNAs (gray lines) were selected in the *VvPDS* exon sequence (black boxes). **(B)** Schematic diagram of the protocol for expression vector construction. **(C)** Schematic diagram of the assembled Cas9/sgRNAs expression vector (pCACRISPR/Cas9) for *Agrobacterium*-mediated suspension-cell transformation.

Four DNA fragments from *VvPDS* exons were cloned and sequenced. We observed that ‘Chardonnay’ and ‘41B’ cultivars had nearly identical *VvPDS* exons to their homologs from the ‘Pinot Noir’ reference genome (PN40024, Supplementary Figure S1). Four 20 bp target sequences with the NGG PAM in *VvPDS* were designed as sgRNA complementary sites. These sgRNA sites were located in the fourth, second, sixth, and seventh exons, capturing 25, 30, 50, 65% GC content, respectively (Table 1). *Arabidopsis* U6 promoter (AtU6) was used to drive expression of the four targets, while CaMV 35S promoter drove *SpCas9* expression. Expression cassettes were inserted into the pCACRISPR/Cas9 binary vector using the homologous recombination method (Ren et al., 2016) (Figure 1).

### Grape Transformation and Identification of Positive Transgenic CMs

**FIGURE 2:**
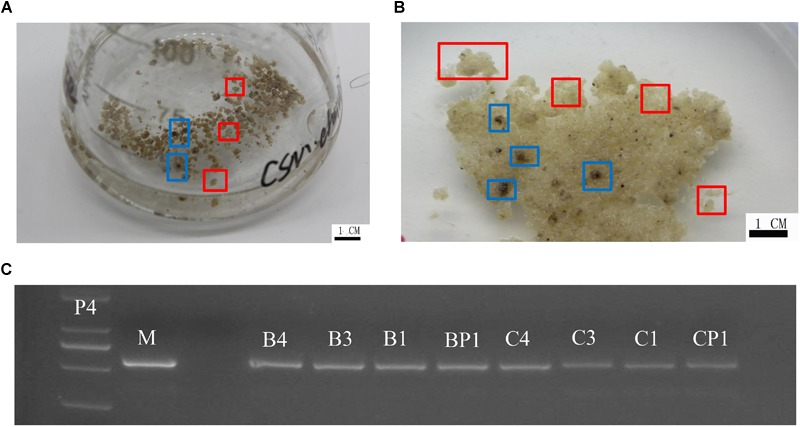
Grape transformation and T-DNA identification. **(A)** Selection of ‘Chardonnay’ cells in liquid medium containing antibiotics. **(B)** Selection of ‘41B’ cells in liquid medium containing antibiotics. Yellowish, resistant transgenic cell masses in red boxes and brown, non-resistant cell masses in blue boxes. **(C)** Identification of exogenous T-DNA insertion in sgRNA-CMs. The PCR template was genomic DNA of sgRNA-CMs, containing specific primers for the hygromycin-resistance gene. Plasmid of the constructed vector (P4) and wild-type DNA (WT) were used as a positive control and a negative control, respectively. T-DNA insertion was detected in sgRNACr1-41B (B1), sgRNACr1-char (C1), sgRNACr3-41B (B3), sgRNACr3-char (C3), sgRNACr4-41B (B4), sgRNACr4-char (C4), sgRNACrP1-41B (BP1), and sgRNACrP1-char (CP1).

‘Chardonnay’ and ‘41B’ cells were transformed with *A. tumefaciens* EHA105 containing the CRISPR/Cas9 vector. After co-culturing for 2 days, inoculated CMs were transferred to media containing antibiotics hygromycin and cefotaxime for 8–10 weeks for screening. Transgenic CM appeared yellow (Figure 2). Successful transformation was validated through PCR with specific primers for hygromycin-resistant gene; eight tested CMs contained the expected exogenous T-DNA insertions (Figure 2 and Supplementary Table S1).

### Detection and Sequencing of Mutations in Transgenic CMs

Every 4 weeks post-agrotransformation, we sequenced DNA fragments containing target sites from eight transgenic sgRNA-PDS CMs (sgRNACr1-char, sgRNACr3-char, sgRNACr4-char, sgRNACrP1-char, sgRNACr1-41B, sgRNACr3-41B, sgRNACr4-41B, sgRNACrP1-41B). After 12 weeks, indels were detected in sgRNACr3-41B and sgRNACr3-char; after 16 weeks, indels were observed in sgRNACr4-char sgRNACr4-41B. Indels for sgRNACr1 and sgRNACrP1 were detected after 20 and 24 weeks in ‘41B’ and in ‘Chardonnay,’ respectively.

**FIGURE 3:**
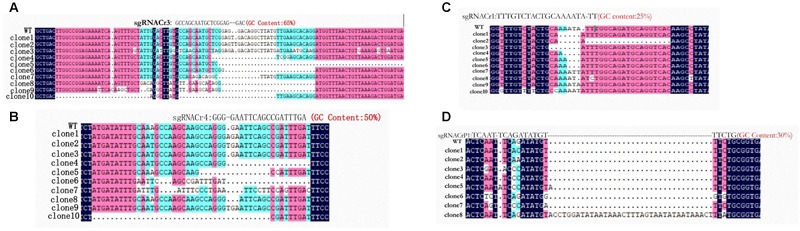
Detection and sequencing of targeted *VvPDS* mutations in transgenic CMs. **(A)** DNA sequences of mutations at target site sgRNACr3 in ‘41B’ (clone 1 to clone 6) and in ‘Chardonnay’ (clone 7 to clone 10). **(B)** Sequence alignment of mutations at target site sgRNACr4 in ‘41B’ (clone 1 to clone 7) and in ‘Chardonnay’ (clone 8 to clone 10). **(C)** Targeted mutagenesis of *VvPDS* at target site sgRNACr1 in ‘41B’ (clone 1 to clone 5) and in ‘Chardonnay’ (clone 5 to clone 10). **(D)** Mutated DNA sequences at target site sgRNACrP1 in ‘41B’ (clone 1 to clone 6) and in ‘Chardonnay’ (clone 7 to clone 8). Homologous nucleotides are shaded, and different colors indicate different homology levels: 100% homology, black; ≥75%, red; ≥50%, blue.

Sanger sequencing showed that fragments from eight sgRNA-CMs (sgRNACr1-char, sgRNACr3-char, sgRNACr4-char, sgRNACrP1-char, sgRNACr1-41B, sgRNACr3-41B, sgRNACr4-41B, sgRNACrP1-41B) contained indels at the target sites (Figure 3). Thus, CRISPR/Cas9 successfully edited four out of six target sites, using four different sgRNAs with 25, 30, 50, and 65% GC content.

### CRISPR/Cas9 Efficiency Using sgRNA Differing in GC Content

We hypothesized that using sgRNA with higher GC content increased CRISPR/Cas9 efficiency, because mutations were detected far more quickly in sgRNACr3-CMs and sgRNACr4-CMs than in sgRNACr1-CMs and sgRNACrP1-CMs. We tested this hypothesis using PCR/RE and T7EI assays.

**FIGURE 4:**
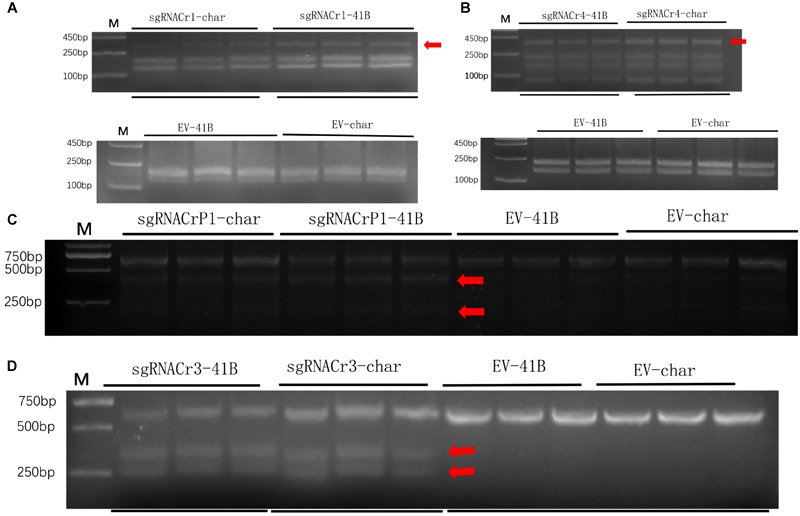
Efficiency of CRISPR/Cas9 using guide RNAs with differing GC content in 41B and “Chardonnay.’ Agarose gels illustrating indel frequency in sgRNACr1-CM lines **(A)** and sgRNACr4-CM lines **(B)** detected using PCR/RE assays of transformed cell masses (red arrowheads indicate mutated bands). Agarose gels showing mutagenesis frequency in sgRNACrP1-CM lines **(C)** and sgRNACr3-CM lines **(D)** detected using T7EI assays of transformed cell masses (red arrowheads indicate cleaved mutated bands). ‘-41B’ labels indel frequency at each target site in ‘41B,’ while ‘-char’ labels indel frequency in ‘Chardonnay.’ WT-41B and WT-char refer to wild-type, untransformed cell masses. Mean indel efficiency was calculated from three independent replicates.

Results from the digestion of PCR products with SspI revealed a band indicative of indels at the target site in sgRNACr1-41B and sgRNACr1-char. Contrary to these results, when PCR products obtained with templates coming from WT-CMs were digested with SspI, there was no additional band, as would be expected when indels are absent. Band intensity suggested that mean indel frequencies of sgRNACr1-41B and sgRNACr1-char were 22.2 and 30.3%, respectively (Figure 4A).

Digestion with EcoRI similarly identified indels in the target sites of sgRNACr4-41B and sgRNACr4-char, whereas no targets in either of WT-CMs had indels (bands) (Figure 4B). Band intensity revealed an average indel frequency of 82.9 and 55.1% for sgRNACr4-41B and sgRNACr4-char, respectively.

Digestion with T7E1 did not yield any additional fragments in WT-CMs, confirming the lack of mutations. However, both sgRNACrP1-41B and sgRNACrP1-char contained indel mutations, as represented by appearance of new bands. Average indel frequency was around 35.4 and 25.6% for the two sgRNACrP1-CMs, respectively (Figure 4C).

Digestion with T7EI also showed CRISPR/Cas9-mutated target sites in sgRNACr3-41B and sgRNACr3-char CMs, but not in WT-CMs (Figure 4D). Average indel frequency for sgRNACr3-41B and sgRNACr3-char was 86.6 and 59.9%, respectively.

Regardless of cultivar, CRISPR/Cas9 editing efficiency dropped significantly when using sgRNA with low GC content (25 and 30%) than those with high GC content (50 and 65%) (Table 2). The highest editing efficiency in both cultivars occurred when we used sgRNA with 65% GC content, suggesting that CRISPR/Cas9 maybe more efficient in the system with higher GC content sgRNA (>50%) than in those with lower GC content sgRNA (<30%). In addition, for sgRNACr1, there is no statistically significant difference between two cultivars. However, under the identical GC contents, sgRNA-41B had a higher mutation rate than sgRNA-char.

**Table 2:** Summary of CRISPR/Cas9-mediated targeted editing efficiency in ‘41B’ and ‘Chardonnay’ grape cultivars.

Cultivars	sgRNACr1 (GC content: 25%)	sgRNACrP1 (GC content: 30%)	sgRNACr4 (GC content: 50%)	sgRNACr3 (GC content: 65%)
41B	30.3 ± 1.7%cd	35.4 ± 8.7%c	82.9 ± 4.6%a	86.6 ± 1.7%a
Char	22.2 ± 3.7%d	25.6 ± 3.7%d	55.1 ± 7.7%b	59.9 ± 6.5%b

*All of the data are the mean values ± SE of three biological replicates. Different letters indicate significant differences between treatments at *P* < 0.05 (Duncan’s multiple range test).*

### *SpCas9* Expression Analysis in ‘41B’ and ‘Chardonnay’ Transgenic Cells

**FIGURE 5:**
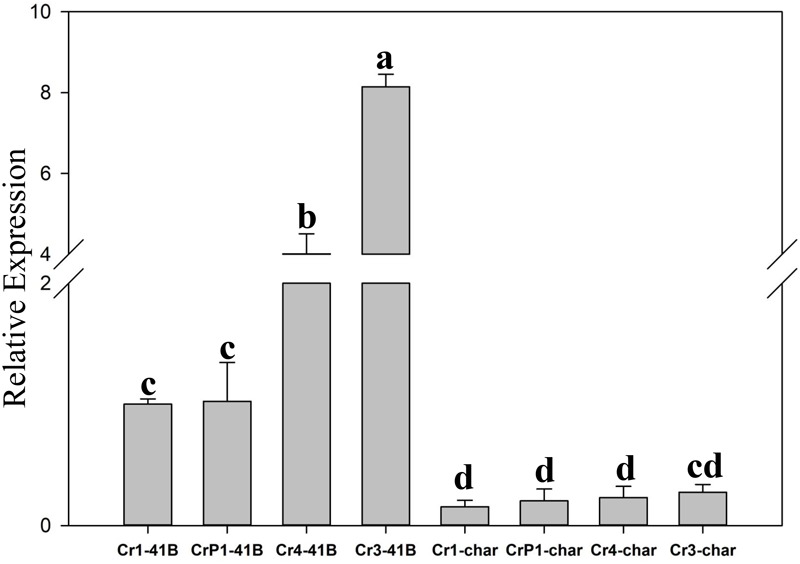
Expression analysis of *Cas9* gene using qRT-PCR. Cas9 expression in sgRNACr1-char (Cr1-char), sgRNACr3-char (Cr3-char), sgRNACr4-char (Cr4-char), sgRNACrP1-char (P1-char), sgRNACr1-41B (Cr1-41B), sgRNACr3-41B (Cr3-41B), sgRNACr4-41B (Cr4-41B), and sgRNACrP1-41B (CrP1-41B). The control was Cr1-41B. Grape *Actin1* was the internal control. Mean expression was calculated from three independent replicates. Vertical bars indicate standard errors of the mean. Different letters indicate significant differences between treatments at *P* < 0.05 (Duncan’s multiple range test).

The results of qRT-PCR revealed that all transgenic sgRNA-CM lines exhibited 35S-promoter-driven *SpCas9* expression (Figure 5). In ‘Chardonnay’ transgenic cells, *SpCas9* expression was detected with different CRISPR/Cas9-induced editing efficiency but no statistically significant difference was found between the four transgenic samples. To make sure the correlation existed between editing efficiency, *SpCas9* expression, and GC content of sgRNA, Pearson’s test analysis was used. In ‘Chardonnay,’ the correlation coefficient (*R*^2^) between editing efficiency and *SpCas9* expression was 0.887 (*P*-value = 0.113); the correlation coefficient (*R*^2^) between editing efficiency and GC content was 0.971 (*P*-value = 0.029). In ‘41B’ transgenic cells, for *SpCas9* expression and GC content, the *R*^2^ coefficient was 0.89 (*P*-value = 0.11) and 0.958 (*P*-value = 0.042), respectively. These results suggested that the GC content of sgRNA, but not *SpCas9* expression level, might be the limiting factor for genome editing.

## Discussion

In the present study, we successfully generated transgenic grapevine in two cultivars through knocking out *VvPDS* with the CRISPR/Cas9 system. As *PDS* gene was widely used in verification of the feasibility of CRISPR/Cas9 system in many species, such as poplar (Fan et al., 2015), potato (Gao et al., 2015), tobacco (Zhang et al., 2016) and watermelon (Tian et al., 2017), we chose *VvPDS* as an effective “tool” gene to study the CRISPR/Cas9 system efficiency in grape. We will use other functional genes to verify the results we found about CRISPR/Cas9 system efficiency in further study. Although the same *VvPDS*-knockout transgenic plants had been generated previously using *V. vini*fera ‘Neo Muscat’ (Nakajima et al., 2017), the authors did not investigate CRISPR/Cas9 efficiency in detail. They reported that the rate of desired mutations remained low even 4 months post-transformation with two sgRNAs that they had designed. We calculated the GC content of their sgRNAs to be 25 and 45%. In this study, we used their sgRNA (PDS-t2) with 25% GC content, along with five other sgRNAs that we designed with the Grape-CRISPR Database^2^ to vary in GC. Four sgRNAs were effective in both ‘Chardonnay’ and ‘41B’ after 24 weeks of transgenic CM selection. Sanger sequencing showed that these four had GC contents of 25, 30, 50, and 65%. Together, our results and previous findings clearly support the hypothesis that CRISPR/Cas9 efficiency is related to sgRNA GC content.

Repair pathways involving NHEJ or HDR introduce small indels at target sites after TGE (Araki and Ishii, 2014). Therefore, indel rates can be used to predict genome editing efficiency (Shan et al., 2014; Tian et al., 2017; LeBlanc et al., 2018). In our study, we calculated mutation rates and CRISPR/Cas9 efficiency based on band intensity observed from gel electrophoresis. Our data led to the conclusion that high GC content in sgRNA increases mutation rate in both ‘Chardonnay’ and ‘41B.’ Our findings corroborate previous research using CRISPR/Cas9 to target *IdnDH* in ‘Chardonnay’ (Ren et al., 2016), where the sgRNA with 65% GC content yielded greater efficiency (higher indel rates) than the sgRNA with 35% GC. Beside our work, in another study it was reported that the mutation efficiency in the T1 (GC content 55%) and T4 (GC content 65%) site targeting *VvWKY58* was 53.63 and 64.91%, respectively (Wang et al., 2018). Additional research suggested that GC content of the target sites also influenced mutation efficiency in tomato: the high editing efficiency (84.00–100.00%) was detected in sgRNAs with GC content above 50%, whereas the sgRNA with GC content containing a relatively low GC content (40%) exhibited lower editing efficiency (72.70%) (Pan et al., 2016). However, even for genes from “41B” cultivar edited with CRISPR/Cas9 system with high GC content sgRNA, it took 12 weeks to detect mutations. We plan to further confirm editing efficiency through investigating whether the ratio of albino (*VvPDS* knockout) plantlets correlates with indel rate and optimize the CRISPR/Cas9 system according to the data we got in a future study.

Grape transgenesis studies typically employ ‘Chardonnay’ and ‘41B’ (Lecourieux et al., 2010; Nicolas et al., 2013, 2014), whereas studies of CRISPR/Cas9 application in grape used ‘Chardonnay’ and ‘Thompson Seedless’ (Wang et al., 2018). In our study, we compared CRISPR/Cas9 efficiency in ‘Chardonnay’ and in ‘41B’ to determine whether grape genotype was an influential factor. Independent of sgRNA GC content, CRISPR/Cas9 efficiency was higher in ‘41B’ than in ‘Chardonnay.’ The results of *SpCas9* expression analysis suggested that *SpCas9* showed different expression level in CRISPR/Cas9 system with different editing efficiency. However, further data analysis showed that statistically significant correlation was found between GC content and CRISPR/Cas9 efficiency in ‘Chardonnay’ and in ‘41B.’ In ‘41B,’ two transgenic samples with high GC content of sgRNA, sgRNACr3-41B and sgRNACr4-41B, showed significantly higher *SpCa9* expression level as well as editing efficiency than others. This result suggested that *SpCas9* expression levels may influence CRISPR/Cas9-induced editing efficiency, but further correlation analysis showed that editing efficiency was more closely related with GC content than *SpCas9* expression levels. Taken together, our data suggest that sgRNA GC content and CM genotype (i.e., cultivar) used for transformation are major limiting parameters governing the efficiency of CRISPR/Cas9-mediated targeted mutagenesis.

## Conclusion

We successfully used CRISPR/Cas9 to knock out *VvPDS* in both ‘Chardonnay’ and ‘41B’ grape cultivars. Our findings supported the hypothesis that sgRNAs with high GC content improved editing efficiency in grapevine. Moreover, we showed that editing efficiency also depends on selecting the appropriate cultivar. Altogether, our study provides valuable data for efforts to optimize the use of CRISPR/Cas9 gene editing in grape.

## Author Contributions

FR performed the experiments and wrote the manuscript. CR and ZZ helped with experiments and data analysis. ZL designed the experiments with the assistance of WD, DL, and SL. DL and ZL reviewed the manuscript. All authors have read and approved the final manuscript.

## Conflict of Interest Statement

The authors declare that the research was conducted in the absence of any commercial or financial relationships that could be construed as a potential conflict of interest.

## Footnotes

1 http://plants.ensembl.org/index.html

2 http://www.grapeworld.cn/pc/design.html
