# Supplementary material for: Efficiency Optimization of CRISPR/Cas9-Mediated Targeted Mutagenesis in Grape
Source: Front Plant Sci. 2019 May 16;10:612. doi: 10.3389/fpls.2019.00612 (PMC6532431; doi:10.3389/fpls.2019.00612)
Supplement: Supplementary file 1 [file Table_1.DOCX]

***Supplementary Materal***

**The Efficiency Optimization of CRISPR/Cas9-mediated Targeted Mutagenesis in Grape**

Fengrui Ren, Chong Ren, Zhan Zhang, Wei Duan, David Lecourieux, Shaohua Li Zhenchang Liang^*^

***Corresponding:** Zhenchang Liang: [ZL249@ibcas.ac.cn](mailto:ZL249@ibcas.ac.cn)

**Table S1 Summary of primer sequence**

| **Primer name** | **Primer sequence (5’→3’)** | **Aims** |
| --- | --- | --- |
| **Cr1-Exon4-F** | ACCATAAAATGATTATGTAATGCAAA | **Amplification of** |
| **Cr1-Exon4-R** | TGTCTTATAATAACCAATAAGGGGAGA | **VvPDS exon** |
| **Cr3-Exon7-F** | GCTTGGATCTCCTAGATGCAGT |  |
| **Cr3-Exon7-R** | TCTAGTTCTACATATGTCGGTG |  |
| **Cr4-Exon6-F** | TGTACTGATCCACCCTATCCTGA |  |
| **Cr4-Exon6-R** | TCCAACCACTGTCACACTGAA |  |
| **CrP1-Exon2-F** | CTGCTGGTGGCTATATGGAGG |  |
| **CrP1-Exon2-R** | TCCACCTGCACCAGCAATTA |  |
| **OligoR1** | GCTATTTCTAGCTCTAAAACAATATTTTGCAGTAGACAAACAATCACTACTTCGACTCT | **Construction of CRISPR-Cas9** |
| **OligoR3** | GCTATTTCTAGCTCTAAAACGTCCTCCGAGCATTGCTGGCAATCACTACTTCGACTCT | **expression vectors** |
| **OligoR4** | GCTATTTCTAGCTCTAAAACGGGGAATTCAGCCGATTTGACAATCACTACTTCGACTCT |  |
| **OligoRP1** | GCTATTTCTAGCTCTAAAACCAGAAACATATCTGAATTGACAATCACTACTTCGACTCT |  |
| **AtU6-F** | CAGGAAACAGCTATGACCATATTCATTCGGAGTTTTTGTATC |  |
| **Actin1-F** | CAGCAGATGTGGATCTCAAA | **qRT-PCR** |
| **Actin1-R** | CTGTGGACAATGGAAGGAC |  |
| **Cas9-F** | AAGCCCATCAGAGAGCAGG |  |
| **Cas9-R** | TGTCGCCTCCCAGCTGAG |  |
| **Hyg-F** | GTCCGTCAGGACATTGTTGGAGCC | **Identification of** |
| **Hyg-R** | GTCTCCGACCTGATGCAGCTCTCGG | **T-DNA** |


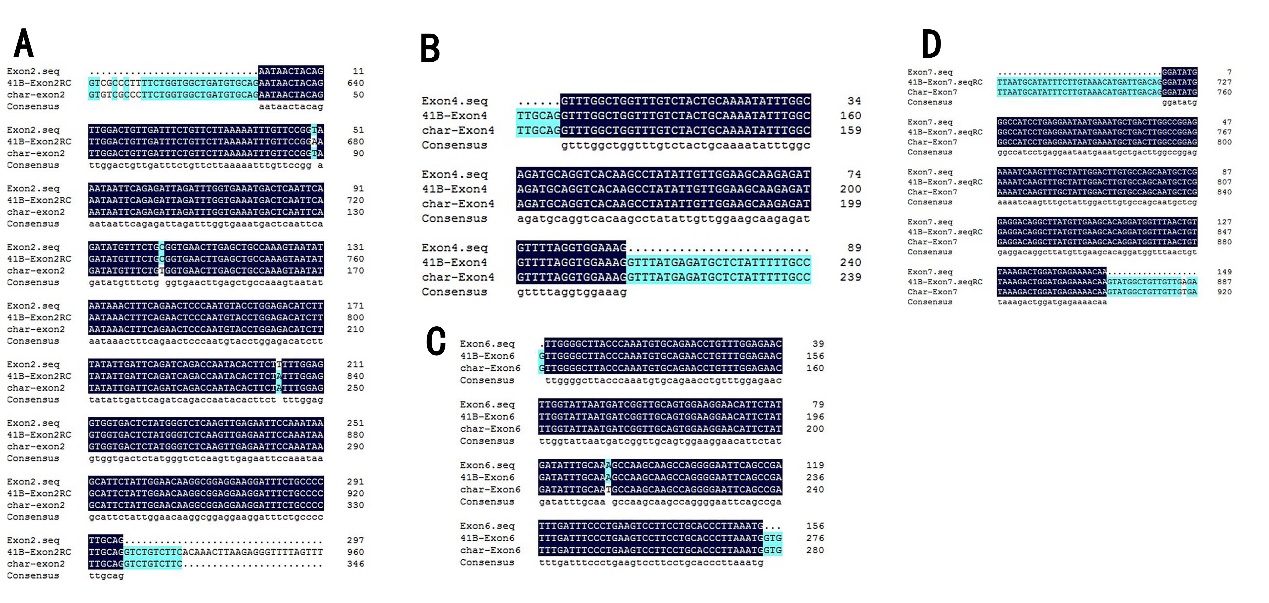


**Figure S1 Comparison of sequencing results of *VvPDS* exons from ‘Chardonnay’ and ‘41B’ SC cultivars (A)-(E)** DNA Sequence alignment of *VvPDS* exon followed by exon 2, exon 4, exon 6 and exon 7. ‘ExonX’ means reference sequences of each *VvPDS* exon in Point Noir. ‘41B-’ and ‘char-’ means DNA sequence of each *VvPDS* exon cloned from ‘41B’ and ‘Chardonnay’ SC. Sequence alignment analysis was performed by using DNAMAN version7.
